# New insights into FtsZ rearrangements during the cell division of *Escherichia coli* from single‐molecule localization microscopy of fixed cells

**DOI:** 10.1002/mbo3.336

**Published:** 2016-02-03

**Authors:** Alexey D. Vedyaykin, Innokentii E. Vishnyakov, Vasilisa S. Polinovskaya, Mikhail A. Khodorkovskii, Anton V. Sabantsev

**Affiliations:** ^1^ Institute of NanobiotechnologiesPeter the Great St. Petersburg Polytechnic UniversityPolytechnicheskaya str. 29Saint Petersburg195251, Russia; ^2^ Institute of CytologyRussian Academy of SciencesTikhoretsky av. 4Saint Petersburg194064, Russia

**Keywords:** Divisome, FtsZ, single‐molecule localization microscopy, Z‐ring

## Abstract

FtsZ – a prokaryotic tubulin homolog – is one of the central components of bacterial division machinery. At the early stage of cytokinesis FtsZ forms the so‐called Z‐ring at mid‐cell that guides septum formation. Many approaches were used to resolve the structure of the Z‐ring, however, researchers are still far from consensus on this question. We utilized single‐molecule localization microscopy (SMLM) in combination with immunofluorescence staining to visualize FtsZ in *Esherichia coli* fixed cells that were grown under slow and fast growth conditions. This approach allowed us to obtain images of FtsZ structures at different stages of cell division and accurately measure Z‐ring dimensions. Analysis of these images demonstrated that Z‐ring thickness increases during constriction, starting at about 70 nm at the beginning of division and increasing by approximately 25% half‐way through constriction.

## Introduction

FtsZ was the first cytoskeleton protein to be identified in prokaryotes (Busiek and Margolin 2015). It is one of the central components of bacterial division machinery and the first protein involved in the assembly of the Z‐ring, a structure that is formed at the middle of dividing cells and guides septation. Mid‐cell FtsZ localization during division was first demonstrated using immunoelectron microscopy (Bi and Lutkenhaus 1991), and later confirmed by fluorescence microscopy (FM) (Levin and Losick 1996; Ma et al. 1996). FtsZ is highly conserved among different species of prokaryotes (Rothfield et al. 1999) and shares homology with eukaryotic tubulin (Nogales et al. 1998). Like tubulin, it possesses GTPase activity and is able to form dynamic filaments. It was shown in vitro that FtsZ can form linear polymers as well as rings and spirals (Mukherjee and Lutkenhaus 1994), but the sizes of these structures are much smaller than the Z‐ring circumference and thus two alternative models were proposed to explain how the Z‐ring is formed from FtsZ protofilaments (Erickson et al. 2010). One of them suggests that the Z‐ring is mostly a disordered array of overlapping short FtsZ filaments, held together by lateral interactions. The second model suggests that short FtsZ protofilaments are organized into longer filaments that can span the whole cell circumference, forming uninterrupted rings or helices. Currently the question of which model accurately describes the in vivo situation is a matter of debate with a number of experimental observations supporting each model.

FtsZ polymerization plays central role in the Z‐ring formation and it is not surprising that this process is tightly controlled to ensure proper septum positioning. In *Escherichia coli*, Min‐system and nucleoid occlusion (NO) system are two main regulators of FtsZ polymerization. Min‐system, composed of MinC, MinD, and MinE proteins, inhibits Z‐ring formation at the cell poles and prevents mini cell formation (Lutkenhaus 2007). The second FtsZ control system – NO – was discovered in a study of nucleoid position influence on division site positioning (Mulder and Woldringh 1989). This mechanism prevents Z‐ring formation over nucleoid that can lead to DNA damage and improper DNA segregation. In *E. coli*, NO is thought to be performed by SlmA protein that binds to certain DNA sequences and inhibits FtsZ polymerization in the proximity of nucleoids (Bernhardt and de Boer 2005).

The majority of studies for the last two decades utilizes fluorescent protein fusions to visualize FtsZ using FM (Ma et al. 1996). This approach has a number of advantages over immunofluorescence, with the ability to perform live‐cell microscopy being one of the main among them. This approach provided a lot of important information about bacterial division mechanisms (Ma et al. 1996; Hale and de Boer 1997; Hu and Lutkenhaus 1999; Wu and Errington 2004; Bernhardt and de Boer 2005; Goehring et al. 2005). Single‐molecule localization microscopy (SMLM) of FtsZ‐mEos2 yielded Z‐ring images with a resolution of about 35 nm (Fu et al. 2010). These results support the model that the Z‐ring is a loose irregular assembly of randomly oriented FtsZ filaments that form rings or small‐pitch helices that are indistinguishable by conventional FM. Later this method was used to analyze FtsZ, ZapA, ZapB, and MatP colocalization in dividing cells, which allowed a model of their arrangement in the Z‐ring to be proposed, suggesting that these proteins form a layered structure, spanning from the cell surface to chromosomes (Buss et al. 2013, 2015).

Despite doubtless advantages, fluorescent fusion protein method has a number of limitations, the majority of them being the fact that protein properties can vary upon addition of a fluorescent domain. This may cause the fusion protein to be unable to fulfill the role of the native protein in the cell. This problem is especially significant for proteins that can form polymers (e.g., FtsZ), since expression of the fusion protein in addition to the native one can cause the shift of equilibrium toward either polymer or monomer form, thus perturbing native conditions. Indeed, it was shown that both N‐terminal and C‐terminal FtsZ‐GFP fusions in *E. coli* are unable to support division in the absence of the native FtsZ (Ma et al. 1996). In the aforementioned article (Fu et al. 2010), authors reported that FtsZ‐mEos2 fusion protein was unable to rescue temperature‐sensitive FtsZ mutant of *E. coli* at the nonpermissive temperature too, and they had to use special conditions (low temperature and minimal medium) to grow cells expressing fusion protein in addition to the native FtsZ. Aside from the possible damage of the native protein, another source of artifacts in fusion protein method is the tendency of many fluorescent proteins to multimerize, which may significantly affect protein localization in bacteria (Landgraf et al. 2012; Wang et al. 2014). One important example of artifacts associated with the use of fluorescent protein fusions are MreB helices in *E. coli* that were reported in the study utilizing MreB‐YFP fusion (Shih et al. 2003). With the use of electron microscopy it was later demonstrated that these helices were an artifact of the particular MreB‐YFP fusion protein (Swulius and Jensen 2012).

It is therefore highly desirable for understanding of mechanisms of FtsZ function in vivo to have data obtained using alternative visualization methods, for instance immunofluorescence microscopy. In recent works (Rowlett and Margolin 2014; Haeusser et al. 2015), immunofluorescence staining was used along with a fluorescent fusion protein to analyze structures formed by FtsZ in *E. coli* using super‐resolution microscopy, specifically 3D structured illumination microscopy (SIM). It was shown that FtsZ has a bead‐like distribution in the Z‐ring, favoring the model of the Z‐ring as an uneven arrangement of unaligned FtsZ filaments. These results agree with SIM data obtained for *Bacillus subtilis* using FtsZ‐GFP fusion (Strauss et al. 2012) and photoactivated localization microscopy (PALM) data obtained with *Caulobacter crescentus* using FtsZ‐Dendra2 fusion (Holden et al. 2014).

The model of the Z‐ring as a loose assembly of randomly oriented short FtsZ filaments was challenged by the work carried out using cryoelectron tomography (cryo‐ET) (Szwedziak et al. 2014). The data obtained suggest that FtsZ forms long uninterrupted filaments at the septum in *E. coli* and *C. crescentus*, which is in a striking disagreement with FM data. Despite the lack of molecular specificity in cryo‐ET and the use of *E. coli* strain B/r H266 that is characterized by unusually thin cells, which could significantly affect the Z‐ring structure, these findings emphasize the need for further study of FtsZ in *E. coli* using high‐resolution techniques.

In the current work SMLM (namely, dSTORM or direct Stochastic Optical Reconstruction Microscopy; Endesfelder and Heilemann 2015) was used in combination with immunofluorescence staining to visualize the native copy of FtsZ at different stages of division process in *E. coli* cells. DNA visualization using intercalating dye helped to accurately interpret FtsZ structures, as septation has to be correlated with DNA segregation process for the cell to produce viable progeny. The achieved resolution (about 25 nm) allowed FtsZ structures to be analyzed in detail and their dimensions to be quite accurately determined. In particular, analysis of correlation between the diameter and thickness of the Z‐ring suggests that it becomes thicker during constriction.

## Experimental Procedures

### Sample preparation for FM

*Esherichia coli* Top10 strain harboring empty pGEX‐4T‐2 plasmid was passaged from the night culture to fresh Luria Bertani medium (LB) supplemented with 100 *μ*g/mL ampicillin and grown at 37°C to OD_600_ ≈ 0.6–0.7. B/r H266 strain was grown in similar way except absence of ampicillin. To produce slow grown Top10 cells, night culture was passaged to M9 medium supplemented with 0.4% glycerol and 100 *μ*g/mL ampicillin and grown at 30°C to OD_600_ ≈ 0.5. Plasmid in Top10 strain was used to prevent contamination by antibiotic resistance selection. Thereafter, bacteria were fixed directly in the medium by adding formaldehyde (Sigma‐Aldrich, St. Louis, MO, USA, final concentration 2.6%), glutaraldehyde (Sigma‐Aldrich, St. Louis, MO, USA, 0.04%), and sodium phosphate buffer (30 mmol/L, pH = 7.4) for 10 min at room temperature, followed by 50 min on ice (Addinall et al. 1996). After fixation, bacteria were harvested by centrifugation and resuspended in PBS with the addition of NaBH_4_ (Sigma‐Aldrich, St. Louis, MO, USA, 1 mg/mL), which reduces cells autofluorescence after glutaraldehyde fixation. After 5 min in NaBH_4_, cells were harvested by centrifugation and resuspended in PBS, harvested again and resuspended in GTE (50 mmol/L glucose, Tris 32.5 mmol/L, pH = 7.5, 10 mmol/L EDTA). Afterward bacteria were immobilized on coverslips coated with poly‐l‐lysine (Sigma‐Aldrich, St. Louis, MO, USA) by allowing cells to settle on the glass surface for 10 min. Polystyrene beads (2.1 *μ*m; Spherotech, Lake Forest, IL, USA) were preliminarily fixed on the coverslip surface to be used as fiduciary markers for the active sample stabilization system based on microsphere position determination using quadrant photodetector.

Cells were permeabilized for 5 min first with a detergent solution (Triton X‐100, 0.1% in PBS) and then for 5 min with lysozyme (10 *μ*g/mL in GTE). After that bacteria were incubated for 30 min in BSA solution (20 mg/mL in PBS) to block nonspecific binding of antibodies. Next, cells were incubated with primary rabbit *α*‐FtsZ antibody (Agrisera, Vännäs, Sweden, AS10715) diluted 1:200 in BSA solution (20 mg/mL in PBS) at 4°C overnight. Then samples were washed thoroughly with detergent solution (0.01% Tween‐20 in PBS). After that cells were incubated with secondary antibodies (F(ab’)2‐fragments of the goat anti‐rabbit antibodies, Alexa 647‐conjugated, Life Technologies, Grand Island, NY, USA A‐21246), in 1:100 dilution in BSA–PBS at room temperature for 1 h. The bacteria were then thoroughly washed again with a Tween‐20 solution. DNA staining was performed by incubating cells in a solution of YOYO‐1 dye (250 nmol/L in PBS) for 10 min.

### FM and data processing

All images were obtained using set up based on motorized microscope AxioImager.Z1 (Carl Zeiss, Oberkochen, Germany), which was described previously (Vedyaykin et al. 2014). Images were acquired using cooled EM‐CCD camera (Andor iXon 897, Belfast, UK) and oil immersion objective with 100× magnification and the numerical aperture of 1.46 (Carl Zeiss, Oberkochen, Germany). One pixel corresponds to 108 nm in the objective focal plane. To detect dyes fluorescence following filter sets were used: Filter Set 10 (Zeiss) for YOYO‐1, and LF635/LP‐B‐000 (Semrock, Rochester, NY, USA) for Alexa 647. Excitation filter from Alexa 647 filter set in some cases was removed to enable the activation of dye molecules using a 405‐nm laser.

Imaging was carried out in PBS‐Tris buffer with pH 7.5, containing 10% w/v glucose, 10 mmol/L 2‐mercaptoethylamine combined with 50 mmol/L 2‐mercaptoethanol, 2 mmol/L cyclooctatetraene, and an oxygen scavenging system (2.5 mmol/L protocatechuic acid and 50 nmol/L protocatechuate 3,4‐dioxygenase) (Olivier et al. 2013a). First conventional fluorescence images of FtsZ and DNA and transmitted light images were obtained using high‐pressure mercury lamp or reduced 635 nm diode laser (in case of removed excitation filter) for fluorescence excitation. To improve signal‐to‐noise ratio series of 20 images were acquired and then averaged. Then Alexa 647 “blinking” was induced by a 635‐nm diode laser with a power density of approximately 1 kW/cm^2^ at the focal plane and 2000–10,000 frames containing individual Alexa 647 molecule images were acquired. Return of Alexa 647 molecules to the fluorescent state was activated by a 405‐nm diode laser if necessary (power density of 0–10 W/cm^2^). During image acquisition sample drift was being compensated by a custom active sample stabilization system: sample displacement was monitored by measuring the position of the bead attached to the coverglass using a quadrant detector and compensated using piezo stage, similar to that described by van Teeffelen et al. (2011).

Imaging was performed using MicroManager (Edelstein et al. 2010). SMLM image reconstruction was performed using ThunderSTORM plugin for ImageJ (Ovesny et al. 2014). Only molecules with intensity of at least 1000 photons and uncertainty in the position of less than 20 nm were included in resulting image. Average localization precision of molecules was about 10 nm; estimated spatial resolution of obtained images was about 25 nm. Resolution evaluation was performed as described previously (Fu et al. 2010). To perform measurements of Z‐ring intensity profiles along lines of 3 pixels in width through reconstructed images were analyzed. To measure Z‐ring width, intensity profile across Z‐ring plane (see Fig. S3, for example) was measured and then approximated by the Gaussian function. Z‐ring width was assumed to be equal to the full width at half maximum of the Gaussian fit. Several width measurements (typically three) were implemented for each Z‐ring. Z‐ring diameter was measured using intensity profile along Z‐ring plane. Diameter was estimated as full width of intensity profile (see Fig. S3, for example).

## Results and Discussion

### SMLM allows Z‐ring parameters at different division stages to be measured accurately

**Figure 1:**
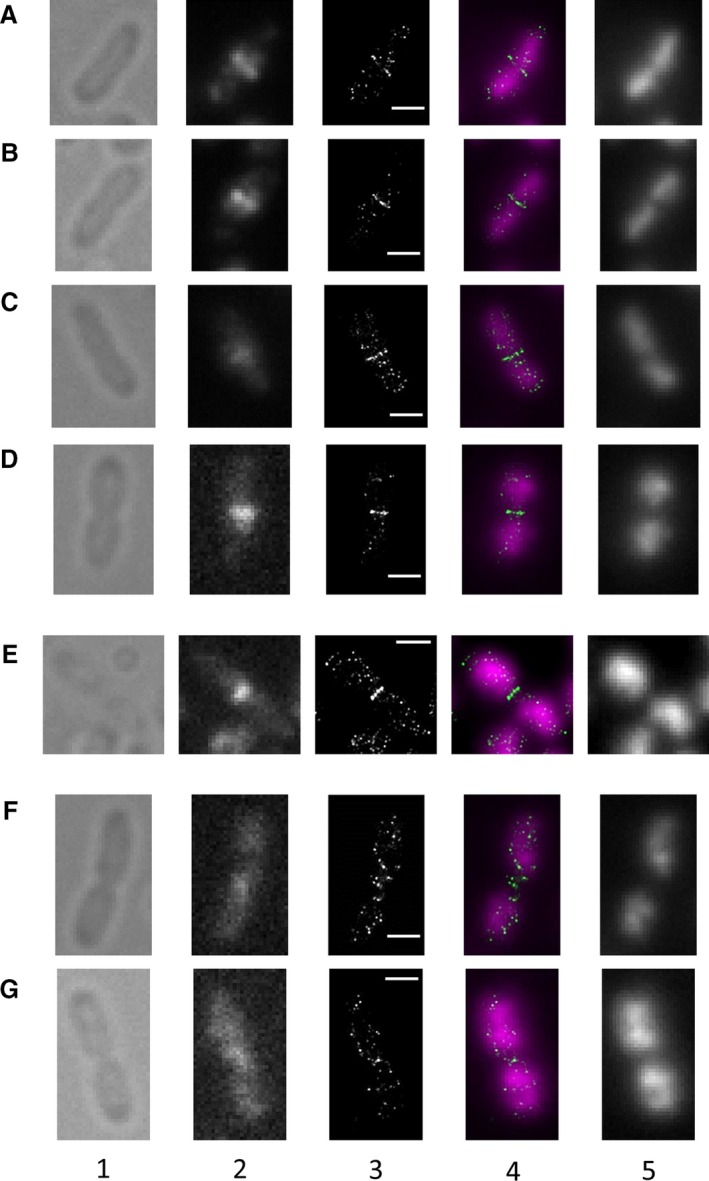
Structures formed by FtsZ in *Esherichia coli* Top10 strain at different stages of the division process under conditions of fast growth. Columns: 1 – transmitted light images, 2 – diffraction‐limited FtsZ images, 3 – single‐molecule localization microscopy (SMLM) FtsZ images, 4 – composite of SMLM FtsZ (green) and diffraction‐limited DNA (magenta) images, 5 – diffraction‐limited DNA images. Rows: A–C – initial division stages prior to septation − the Z‐ring formation, D and E – septum constriction and the Z‐ring thickening, F and G – final stages of division and disassembly of the Z‐ring. Scale bar corresponds to 1 *μ*m.

SMLM combined with indirect immunofluorescent staining was used to visualize FtsZ structures in dividing *E. coli* cells with DNA visualized by intercalating dye YOYO‐1. It is known that immunofluorescence sample preparation could introduce artifacts, especially at the SMLM resolution level (Whelan and Bell 2015), however certain degree of reassurance comes from reports that no significant FtsZ fixation artifacts were observed by SMLM (Fu et al. 2010) and SIM (Rowlett and Margolin 2014). To achieve the aim of the study, recently proposed protocol for SMLM with Alexa 647 dye in the buffer containing PCA‐PCD oxygen scavenging system (protocatechuic acid and protocatechuat – 3,4‐dioxygenase) and 2 mmol/L cyclooctatetraene was used (Olivier et al. b). Typically from 500 to several thousands of Alexa 647 molecules were detected per cell. Mid‐cell FtsZ localization was observed in 86% of Top10 cells in exponentionally growing (fast growth conditions – 37°C, LB medium) culture (OD_600_ ≈ 0.6), about 3% of cells demonstrated filamentous morphology (some of them had more than 2 nucleoids with FtsZ localized in‐between), and the rest demonstrated no pronounced FtsZ localization (possibly these cells underwent FtsZ rearrangement between previous and next septations). In most fast‐growing Top10 cells a bit more then a third of FtsZ molecules was concentrated in a thin structure at the mid‐cell (mean ± SD is 37 ± 9%, *N* = 67). Using combined FtsZ‐SMLM + DNA images (column 4 on Fig. 1), cells with visible FtsZ localization in the middle could be sorted into three main classes according to their division stage with DNA providing independent information on cell cycle progression: early stage – start of division when Z‐ring is formed at the mid‐cell and DNA is not completely segregated (Fig. 1A–C and Fig. S1A), Z‐ring diameter D > 900 nm; middle stage – septum contraction (Fig. 1D and E and Fig. S1B–D), 500 < D < 900 nm and late stage – Z‐ring contraction into a tight spot (Fig. 1F and G and Fig. S1E–I), D < 500 nm, and its disassembly supposedly followed by preparation for the next division. According to obtained images, fast‐growing Top10 cells were distributed between three stages of cytokinesis as follows: early stage – 36%, middle stage – 55%, and late stage – 9% (total number of cells *N* = 242, see Fig. S4 as an example of fields with cells).

Z‐ring dimensions are beyond the resolution of conventional fluorescence and even SIM microscopy (Rowlett and Margolin 2014). Our approach allowed Z‐ring dimensions to be measured with high precision (measurement method is explained on Fig. S3). Average of observed Z‐ring axial thickness was 74 ± 15 nm (mean ± SD) for fast‐growing Top10 strain (total number of measured cells *N* = 67). Average axial thickness of the Z‐ring in *E. coli* was previously reported (Buss et al. 2015) to be 115 nm, possibly reflecting the formation of denser structures when fluorescent FtsZ fusion is expressed in addition to the native protein due to the shift in polymerization equilibrium or alteration of FtsZ properties in the fusion protein, which was unable to support division in the absence of the native FtsZ. Another possible explanation for this distinction may be the difference in growth conditions; in the aforementioned work bacteria were cultured under slow growth conditions (room temperature and M9 medium), whereas in our work bacteria were cultured under fast growth conditions (37°C and LB medium). We hypothesized that growth conditions could influence the structures formed by FtsZ, as it significantly affects cell size and the division process (Mannik and Bailey 2015).

To test whether growth conditions and strain used could influence the measured parameters, we performed similar experiment on the same *E. coli* strain (Top10) under slow growth conditions (30°C in M9 medium) and on B/r H266 strain under fast growth conditions. Cells in both cases demonstrated FtsZ localization patterns which are similar to fast‐growing Top10 cells (see Figs. S5 and S6 as examples of fields with cells). Z‐ring thickness under slow growth conditions in Top10 strain was measured to be 77 ± 16 nm (*N* = 40), thus we observed no substantial difference between Z‐ring thicknesses in different growth conditions. Therefore, it seems more plausible that difference between data obtained using PALM (115 nm) and in current study (about 75 nm) is not due to growth conditions, but is more likely related to the use of fusion protein. *Escherichia coli* strain B/r H266 was reported to form continuous FtsZ filament in septum under conditions of slow growth (Szwedziak et al. 2014). The data obtained using B/r H266 under fast growth conditions show that Z‐ring has similar thickness as in Top10 – 80 ± 17 nm (*N* = 50). These data together show that FtsZ in *E. coli* forms Z‐rings with quite conservative thickness which does not significantly depend on either growth conditions or strain features. Remarkably, observed Z‐ring axial thickness is very close to the axial thickness of *C. crescentus* Z‐ring (Holden et al. 2014) measured using SMLM (71 nm).

One of the important questions about the Z‐ring organization is whether FtsZ forms a continuous filament at the septum or FtsZ protofilaments are organized into a more loose arrangement with various FtsZ density and perhaps protofilament orientation. A number of studies utilizing various FM techniques such as PALM, SIM, and fluorescence polarization microscopy (Strauss et al. 2012; Si et al. 2013; Holden et al. 2014; Rowlett and Margolin 2014; Haeusser et al. 2015) support the latter model. At the same time, cryo‐ET yielded strikingly different results (Szwedziak et al. 2014): formation of long continuous filaments that span the whole cell circumference was observed at the septum of dividing *E. coli* and *C. crescentus*. Authors interpreted these structures as ordered arrangements of aligned FtsZ filaments. This observation is in clear contradiction with the data obtained for *E. coli* using different FM techniques. It is important to note that cryo‐ET lacks molecular specificity and the composition of observed filaments had to be inferred from indirect evidence. Our images, especially the ones where Z‐ring is seen at an angle (Fig. S2), demonstrate uneven FtsZ distribution throughout the Z‐ring. However, it is hard to rule out the possibility that this observed irregularity is due to the stochastic nature of localization microscopy, since indirect immunofluorescence method lacks one‐to‐one correspondence between protein molecules and fluorescence localizations due to a number of potential amplification steps.

### Z‐ring thickens during constriction

**Figure 2:**
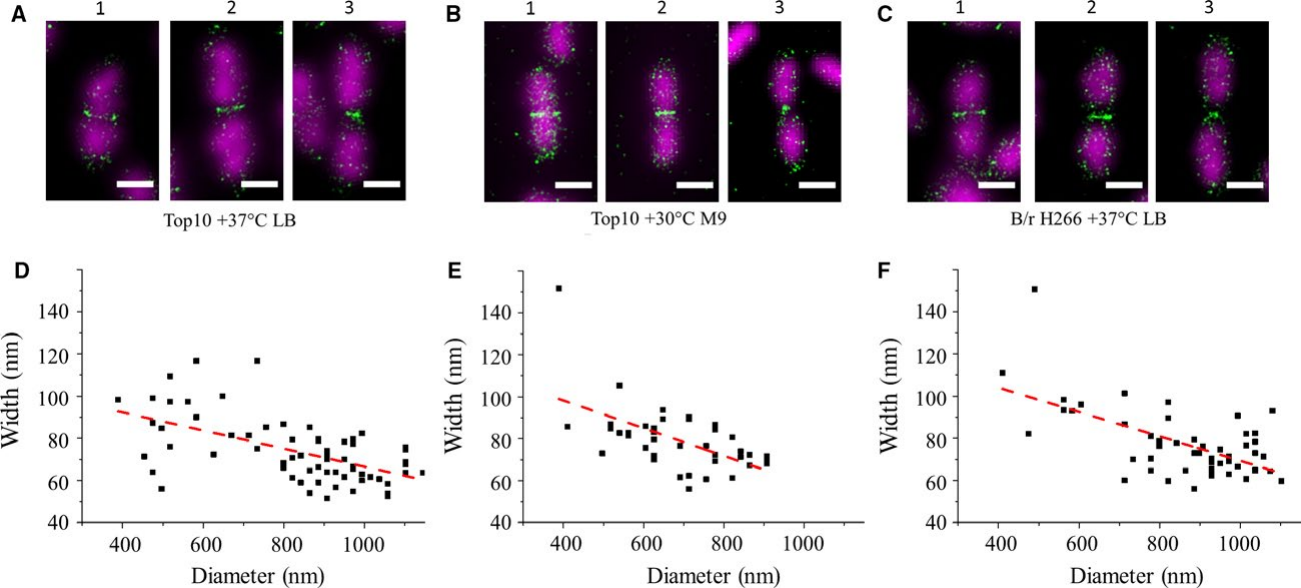
Correlation between Z‐ring diameter and width. A, D – Top10 strain in fast growth conditions. Red dashed line on panel D corresponds to linear regression of scatter plot data *y* = *ax* + *b*, where *a* = −(43 ± 8)·10^−3^ (mean ± SD), *b* = 109 ± 7 nm. B, E – Top10 strain in slow growth conditions. Red dashed line on panel E corresponds to linear regression of scatter plot data *y* = *ax* + *b*, where *a* = −(66 ± 16)·10^−3^, *b* = 125 ± 11 nm. C, F – B/r H266 strain in fast growth conditions. Red dashed line on panel F corresponds to linear regression of scatter plot data *y* = *ax* + *b*, where *a* =−(58 ± 11)·10^−3^, *b* = 127 ± 10 nm. Panels 1, 2, 3 on A–C represent cells at early, middle, and late constriction stages, respectively. Only those cells in which FtsZ was localized as a regular‐shaped band at the mid‐cell were included in this analysis. Scale bar corresponds to 1 *μ*m.

Our measurements using fast grown Top10 cells demonstrated a significant correlation between Z‐ring diameter and axial thickness with the latter increasing with decreasing diameter (Pearson's correlation coefficient −0.57, *n* = 67, *P* < 0.0001, for statistical significance of the correlation) (Fig. 2A and D). At early division stages Z‐ring has a diameter of about 900–1000 nm and is approximately 70‐nm thick. Half‐way through septum constriction apparent Z‐ring axial thickness increases on average by approximately 25%. The similar situation takes place in slow‐growing Top10 (Fig. 2B and E) and fast‐growing B/r H266 (Fig. 2C and F) (Pearson's correlation coefficients −0.64 and −0.60, *n* = 40 and 50, respectively, *P* < 0.0001 for statistical significance of the correlation in both cases). This observation may indicate rearrangement of FtsZ filaments in Z‐ring during constriction. These data are consistent with experimental results and the model of force generation by FtsZ, reported previously (Lan et al. 2009). Also it is in agreement with data obtained on a coccoid bacterium (Jacq et al. 2015), where the dimensions of Z‐rings in *Streptococcus pneumoniae* were measured using PALM (Jacq et al. 2015). The Z‐ring in this diverse bacterial species demonstrates greater thickness (95 and 127 nm for early and late division stages, respectively) than Z‐ring in *E. coli* measured in current study. Authors found no change in total number of FtsZ molecules in the Z‐ring during constriction and concluded that Z‐ring thickening is not due to recruitment of more FtsZ molecules and constriction does not rely on loss of FtsZ molecules. Study on live *B. subtilis* cells expressing FtsZ‐GFP fusion demonstrated no change in total fluorescence at the septum during constriction (Strauss et al. 2012). To investigate the possible Z‐ring thickening and constriction mechanisms we analyzed the correlation between Z‐ring diameter and portion of FtsZ molecules at the septum (Fig. S7). We found that portion of FtsZ molecules at the septum slightly decreases upon Z‐ring constriction, but this effect is less statistically significant (Pearson's correlation coefficient 0.32, *n* = 67, *P* < 0.004, for statistical significance of the correlation) then thickening of the Z‐ring and should be taken into account with caution. Anyway, this observation rules out additional recruitment of FtsZ molecules as a possible cause of the Z‐ring thickening and leaves open the question of FtsZ molecules loss as a mechanism of septum constriction. It would be desirable to follow the constriction of the Z‐ring in live cells with subdiffractional resolution to clarify these questions. Interestingly, no correlation between axial thickness and diameter of the Z‐ring was observed in *C. crescentus* (Holden et al. 2014), possibly indicating differences in constriction mechanisms.

## Conclusion

SMLM in combination with immunofluorescence staining was used to visualize native FtsZ in *E. coli* cells under fast and slow growth conditions with resolution well below the diffraction limit. This approach allowed us to obtain images of FtsZ structures at different stages of cell division. High resolution of this method (about 25 nm in *x*–*y* plane) allowed structures formed by native FtsZ in bacterial cells to be analyzed at the level beyond the reach of conventional FM. Obtained data suggest that during the cytokinesis Z‐ring gradually constricts and thickens, and is eventually dismantled, with FtsZ possibly distributed between daughter cells.

Comparison of results obtained using different approaches mentioned above, including results presented in this study, allows us to conclude that the question of Z‐ring structure is still far from consensus, and further research is needed to draw definitive conclusions. One of the possible sources of valuable new information could be high‐resolution 3D visualization of divisome components using 3D SMLM in combination with immunofluorescence staining. Structural information obtained by a different super‐resolution technique (namely, STED – STimulated Emission Depletion microscopy) would also be beneficial. In general, due to the fact that every visualization technique can introduce artifacts of its own, it is important to compare data obtained using different methods to obtain an accurate understanding of the Z‐ring structure and bacterial division mechanisms in general.

This study demonstrates the versatility of SMLM and immunofluorescence combination that provides extensive opportunities to visualize different cell components. Experimental protocol can be fairly easily extended to incorporate SMLM visualization of the cell wall, an additional protein or nucleic acids using fluorescence in situ hybridization (Vedyaykin et al. 2015). Visualization protocol developed in this study can be further utilized to investigate cytokinesis regulation mechanisms, such as NO and minCDE system, as well as FtsZ rearrangements during SOS response and effects of different mutations in FtsZ and other divisome components on the structure of Z‐ring.

## Conflict of Interest

None declared.

## Supporting information


**Figure S1.** Combined SMLM FtsZ (green) and diffraction‐limited DNA (magenta) images of *Escherichia coli* Top10 cells at different stages of division. Images are positioned from A to I roughly according to the septation progress: initial Z‐ring formation stage (A), Z‐ring constriction (B–D), and final stages of division (E–I). Scale bar corresponds to 1 *μ*m.
**Figure S2.** Combined SMLM FtsZ (green) and diffraction‐limited DNA (magenta) images of tilted *Escherichia coli* Top10 cells showing Z‐rings at different stages of division. These images provide good illustration of observed Z‐ring thickening during constriction. Images are positioned from A to J roughly according to the division progress. Scale bar corresponds to 1 *μ*m.
**Figure S3.** Description of Z‐ring dimensions measurement procedure. A, C – measurement of Z‐ring diameter. To estimate Z‐ring diameter, the intensity profile of the line of 3 pixels in width along Z‐ring plane (yellow dotted line on panel A) was measured. The diameter was taken to be equal to full width of intensity profile of this line. B, D – measurement of Z‐ring width. To estimate Z‐ring width the intensity profile of the line of 3 pixels in width across Z‐ring plane (yellow dotted line on panel B) was measured. The width was taken to be equal to FWHM of intensity profile of this line. Several measurements of width in different positions (usually three positions) of each Z‐ring were performed to reduce inaccuracy; obtained values were averaged.
**Figure S4.** A, B – Combined SMLM FtsZ (green) and diffraction‐limited DNA (magenta) images of two different fields of view with *Escherichia coli* Top10 cells in fast growth conditions (37°C and LB medium). The fields show that most of cells at the moment of fixation were on stages of Z‐ring formation or its constriction. Only a few cells were on final stage of septation. Those cells which showed regular bands in the middle were used to perform Z‐ring measurements.
**Figure S5.** A, B – Combined SMLM FtsZ (green) and diffraction‐limited DNA (magenta) images of two different fields of view with *Escherichia coli* Top10 cells in slow growth conditions (30°C and M9 medium). Those cells which showed regular bands in the middle were used to perform Z‐ring measurements.
**Figure S6.** A, B – Combined SMLM FtsZ (green) and diffraction‐limited DNA (magenta) images of two different fields of view with *Escherichia coli* cells (B/r H266 strain in fast growth conditions – 37°C and LB medium). Cells demonstrated Z‐rings at different stages of division. The fields show that most of cells at the moment of fixation were on stages of Z‐ring formation or its constriction. Only a few cells were on final stage of septation. Those cells which showed regular bands in the middle were used to perform Z‐ring measurements.
**Figure S7.** Portion of FtsZ molecules at the septum versus Z‐ring diameter scatter plot (fast‐growing Top10 cells were analyzed). Each dot corresponds to a single cell. Red dashed line corresponds to linear regression of scatter plot data *y* = *ax* + *b*, where *a* = −(1.4 ± 0.5) • 10^−4^ nm^−1^ (mean ± SD), *b* = 0.25 ± 0.04. Pearson's correlation coefficient 0.32 (*n* = 67), *P* < 0.004 for statistical significance of the correlation.
