# Supplementary material for: New insights into FtsZ rearrangements during the cell division of Escherichia coli from single‐molecule localization microscopy of fixed cells
Source: Microbiologyopen. 2016 Feb 3;5(3):378–86. doi: 10.1002/mbo3.336 (PMC4905991; doi:10.1002/mbo3.336)
Supplement: Supplementary file 1 — Figure S1. Combined SMLM FtsZ (green) and diffraction‐limited DNA (magenta) images of Escherichia coli Top10 cells at different stages of division. Images are positioned from A to I roughly according to the septation progress: initial Z‐ring formation stage (A), Z‐ring constriction (B–D), and final stages of division (E–I). Scale bar corresponds to 1 μm. Figure S2. Combined SMLM FtsZ (green) and diffraction‐limited DNA (magenta) images of tilted Escherichia coli Top10 cells showing Z‐rings at different stages of division. These images provide good illustration of observed Z‐ring thickening during constriction. Images are positioned from A to J roughly according to the division progress. Scale bar corresponds to 1 μm. Figure S3. Description of Z‐ring dimensions measurement procedure. A, C – measurement of Z‐ring diameter. To estimate Z‐ring diameter, the intensity profile of the line of 3 pixels in width along Z‐ring plane (yellow dotted line on panel A) was measured. The diameter was taken to be equal to full width of intensity profile of this line. B, D – measurement of Z‐ring width. To estimate Z‐ring width the intensity profile of the line of 3 pixels in width across Z‐ring plane (yellow dotted line on panel B) was measured. The width was taken to be equal to FWHM of intensity profile of this line. Several measurements of width in different positions (usually three positions) of each Z‐ring were performed to reduce inaccuracy; obtained values were averaged. Figure S4. A, B – Combined SMLM FtsZ (green) and diffraction‐limited DNA (magenta) images of two different fields of view with Escherichia coli Top10 cells in fast growth conditions (37°C and LB medium). The fields show that most of cells at the moment of fixation were on stages of Z‐ring formation or its constriction. Only a few cells were on final stage of septation. Those cells which showed regular bands in the middle were used to perform Z‐ring measurements. Figure S5. A, B – Combined SMLM FtsZ (green) and [file MBO3-5-378-s001.pdf]

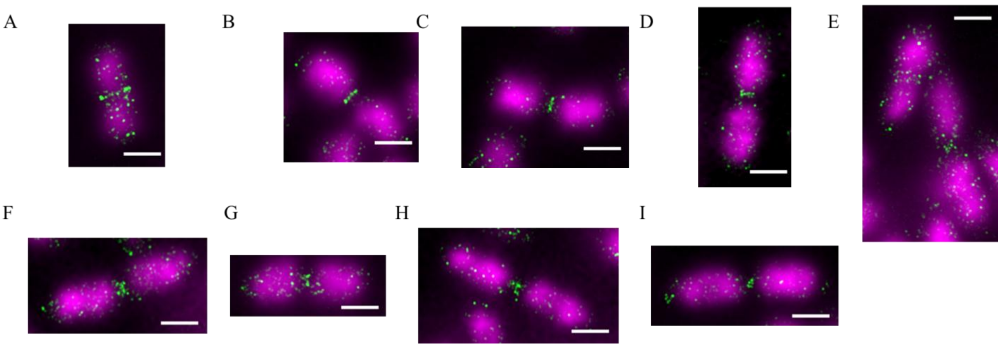

Figure S1. Combined SMLM FtsZ (green) and diffraction-limited DNA (magenta) images of *E.coli* Top10 cells at different stages of division. Images are positioned from A to I roughly according to the septation progress: initial Z-ring formation stage (A), Z-ring constriction (B-D) and final stages of division (E-I). Scale bar corresponds to 1 µm.

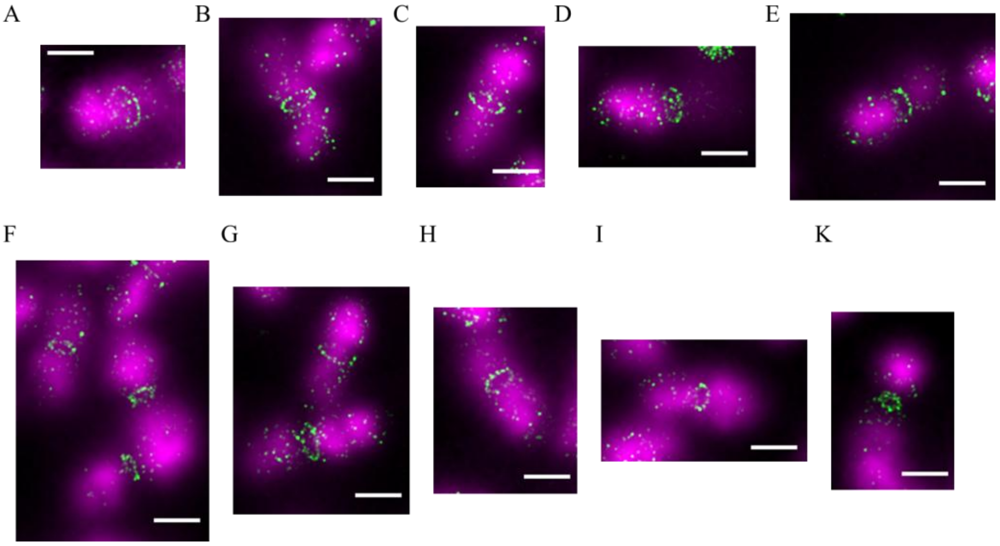

Figure S2. Combined SMLM FtsZ (green) and diffraction-limited DNA (magenta) images of tilted *E.coli* Top10 cells showing Z-rings at different stages of division. These images provide good illustration of observed Z-ring thickening during constriction. Images are positioned from A to K roughly according to the division progress. Scale bar corresponds to 1 µm.

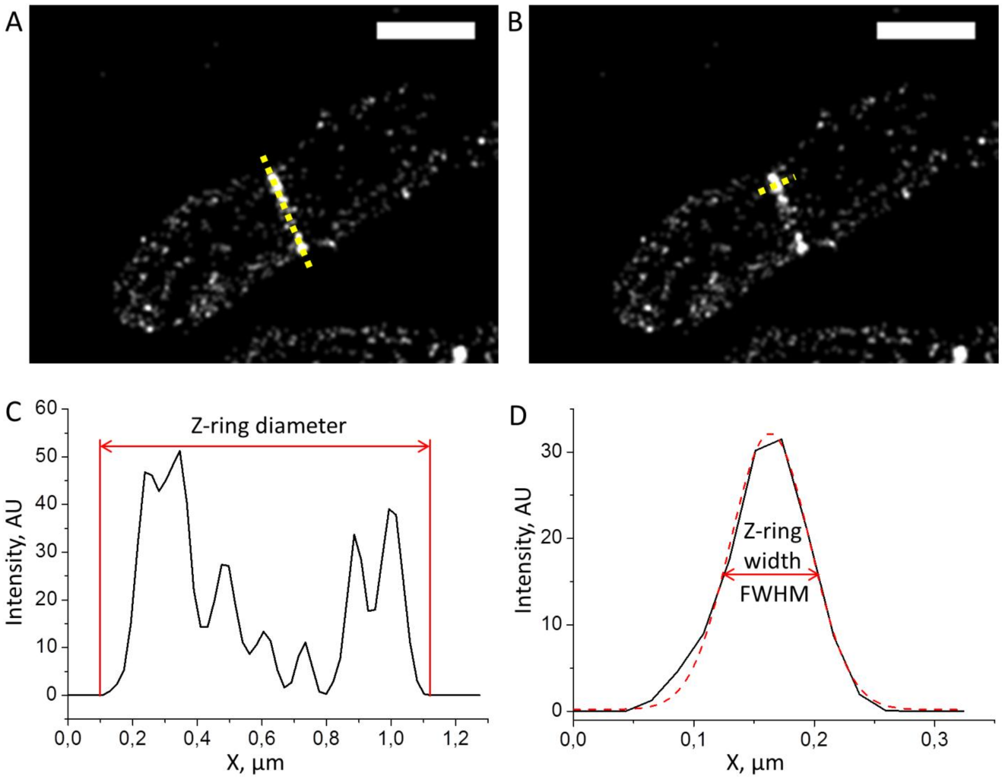

Figure S3. Description of Z-ring dimensions measurement procedure. A, C – measurement of Z-ring diameter. To estimate Z-ring diameter, the intensity profile of the line of 3 pixels in width along Z-ring plane (yellow dotted line on panel A) was measured. The diameter was taken to be equal to full width of intensity profile of this line. B, D - measurement of Z-ring width. To estimate Z-ring width the intensity profile of the line of 3 pixels in width across Z-ring plane (yellow dotted line on panel B) was measured. The width was taken to be equal to FWHM of intensity profile of this line. Several measurements of width in different positions (usually 3 positions) of each Z-ring were performed to reduce inaccuracy; obtained values were averaged.

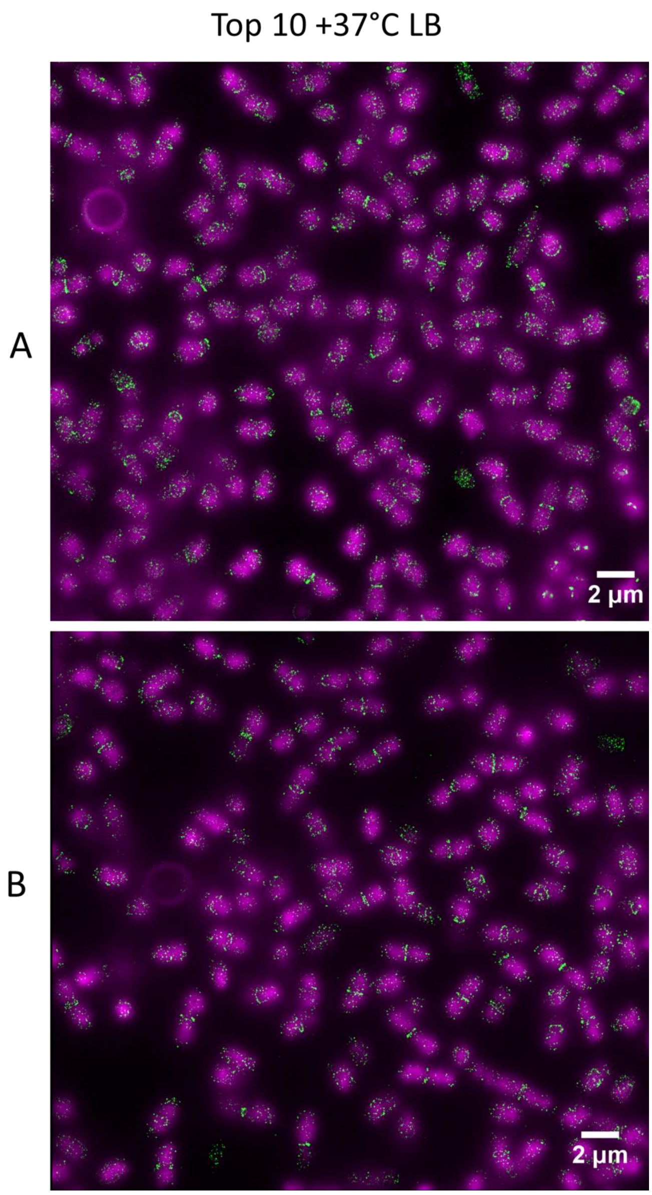

Figure S4. A,B: combined SMLM FtsZ (green) and diffraction-limited DNA (magenta) images of two different fields of view with *E.coli* Top10 cells in fast growth conditions (37 °C and LB medium). The fields show that most of cells at the moment of fixation were on stages of Z-ring formation or its constriction. Only a few cells were on final stage of septation. Those cells which showed regular bands in the middle were used to perform Z-ring measurements.

Top10 +30°C M9

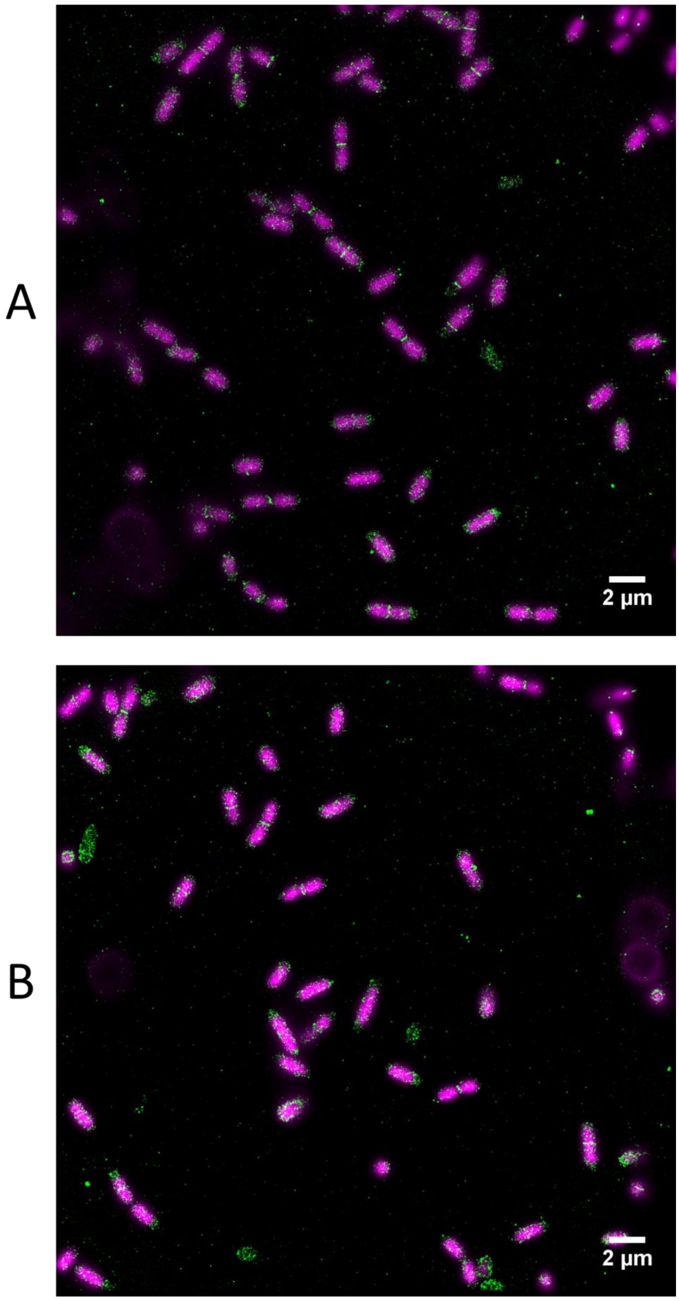

Figure S5. A, B: combined SMLM FtsZ (green) and diffraction-limited DNA (magenta) images of two different fields of view with *E.coli* Top10 cells in slow growth conditions (30 °C and M9 medium). Those cells which showed regular bands in the middle were used to perform Z-ring measurements.

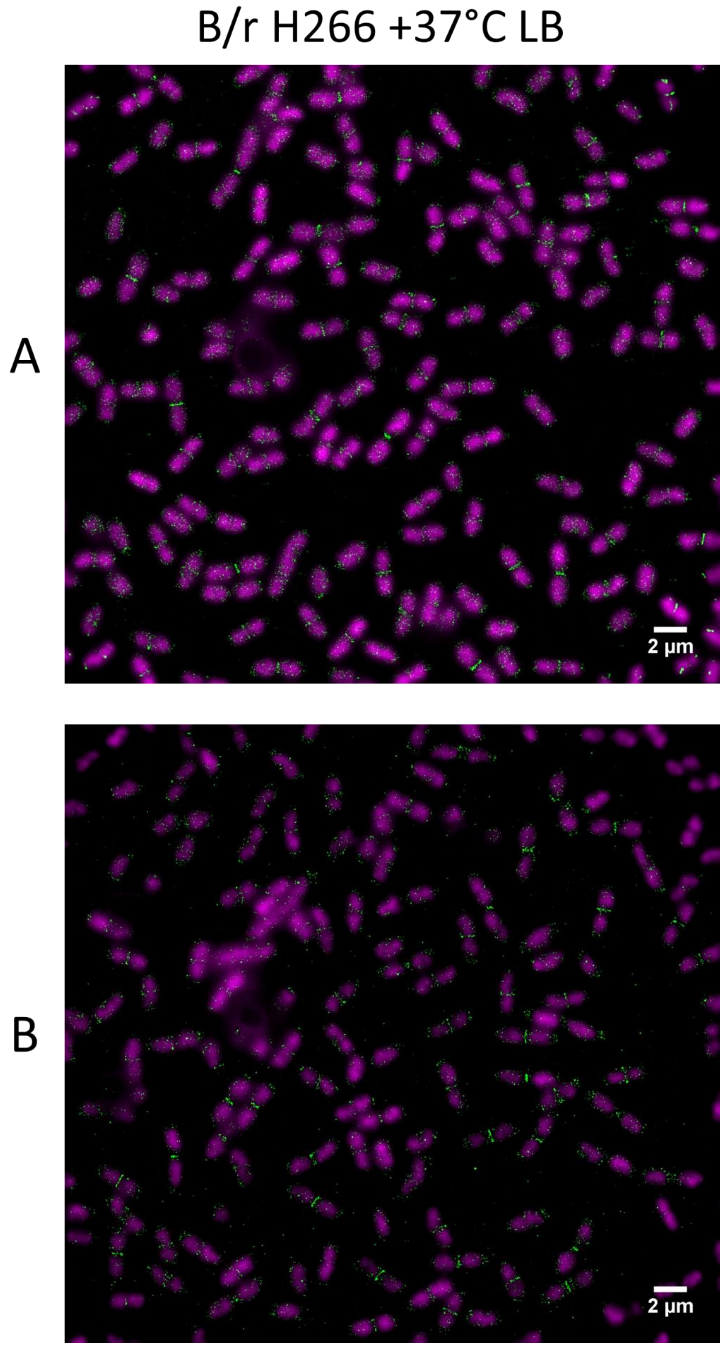

Figure S6. A,B: combined SMLM FtsZ (green) and diffraction-limited DNA (magenta) images of two different fields of view with *E.coli* cells (B/r H266 strain in fast growth conditions- - 37 °C and LB medium). Cells demonstrated Z-rings at different stages of division. The fields show that most of cells at the moment of fixation were on stages of Z-ring formation or its constriction. Only a few cells were on final stage of septation. Those cells which showed regular bands in the middle were used to perform Z-ring measurements.

Отформатировано: английский (США)

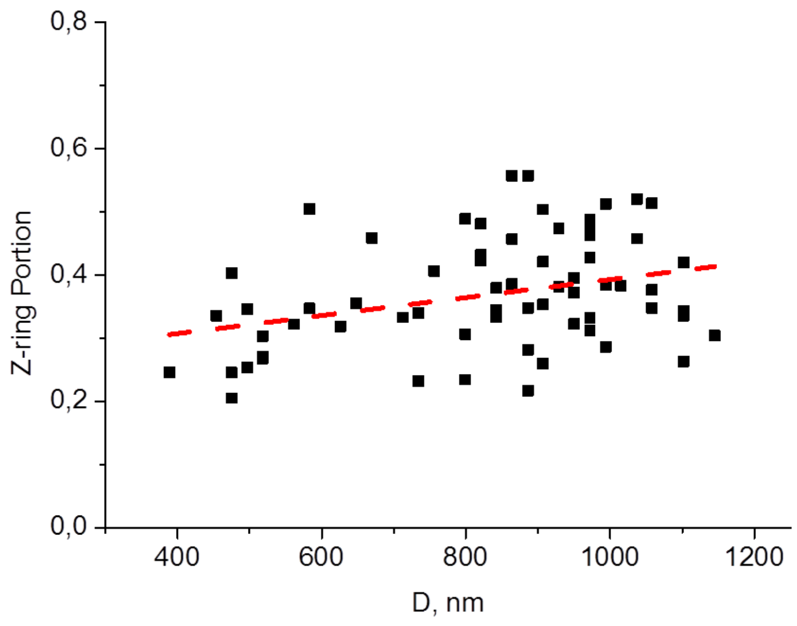

Figure S7. Portion of FtsZ molecules at the septum vs Z-ring diameter scatter plot (fast-growing Top10 cells were analyzed). Each dot corresponds to a single cell. Red dashed line corresponds to linear regression of scatter plot data y= ax+b, where a= -(1.4±0.5)•10^{-4} nm^{-1} (mean±SD), b=0.25±0.04. Pearson's correlation coefficient 0.32 (n=67), p<0.004 for statistical significance of the correlation.
